# Supplementary material for: From Complex Interventions to Complex Systems: Using Social Network Analysis to Understand School Engagement with Health and Wellbeing
Source: Int J Environ Res Public Health. 2019 May 14;16(10):1694. doi: 10.3390/ijerph16101694 (PMC6571883; doi:10.3390/ijerph16101694)
Supplement: Supplementary file 1 [file ijerph-16-01694-s001.pdf]

Table S1. Job role key for Highbridge School net-maps

Node number	Job role	Node number	Job role
**1**	Chair of governors	**14**	School nurse
**2**	Safeguarding officer	**15**	Teaching assistants
**3**	Head teacher	**16**	All students
**4**	Deputy head teacher	**17**	All parents
**5**	Healthy Schools Coordinator	**18**	Promoting inclusion officer
**6**	All staff (daily meeting)	**19**	Women's aid
**7**	All staff (bulletin)	**20**	SANDS (drug/alcohol misuse)
**8**	Healthy living team (multi-agency)	**21**	YAS clinic (sexual health)
**9**	Wellbeing manager	**22**	NHS (many services)
**10**	Communities first staff	**23**	Team Around the Family (TAF)
**11**	Technicians (Science/Food/Art)	**24**	Social services
**12**	Local doctor's surgery	**25**	Youth service
**13**	Youth workers		

Table S2. Job role key for Oakwood School net-maps

**Node number**	**Job Role**	**Node number**	**Job Role**
**1**	Head teacher	**17**	Parents
**2**	Deputy head	**18**	Fire service
**3**	Assistant head 1	**19**	Barnardos
**4**	Assistant head 2	**20**	Women's aid
**5**	Assistant head 3	**21**	Drug/alcohol agencies
**6**	Office manager	**22**	Counsellor
**7**	All teaching staff	**23**	Young carers
**8**	Head of PSE	**24**	Educational welfare officer
**9**	Additional learning needs coordinator	**25**	Youth offending team
**10**	Heads of year	**26**	School health nurse
**11**	School nurse	**27**	Looked after children team
**12**	Non-teaching staff x35	**28**	Police
**13**	Speech/language support officer	**29**	Inclusion service
**14**	Emotional literacy support assistant	**30**	Educational psychologist
**15**	School counsellor	**31**	Child and Adolescent Mental Health Service (CAMHS)
**16**	Students	**32**	Social services

Table S3. Job role key for Woodlands School net-map

Node number	Job Role	Node number	Job Role
**1**	Head teacher	**17**	Canteen staff (x14)
**2**	Assistant head 1	**18**	Dining toom assistants
**3**	Assistant head 2	**19**	Learning support assistants
**4**	Assistant head 3	**20**	School council members
**5**	Assistant head 4	**21**	All parents
**6**	Deputy head 1	**22**	All year groups
**7**	Deputy head 2	**23**	Governing body
**8**	Heads of year (x5)	**24**	School nurses (x2)
**9**	Science teacher/ttudent voice	**25**	Drug aid
**10**	Food technology teacher	**26**	Social services
**11**	Girls' PE teacher	**27**	Child and Adolescent Mental Health Services (CAMHS)
**12**	PE head of department	**28**	Mind (mental health charity)
**13**	PE teacher	**29**	Shelter Cymru
**14**	Office staff (x6)	**30**	Safer Area
**15**	Caretakers	**31**	Healthy Schools Coordinator
**16**	Cleaners		

Table S4. Job role key for Greenfield School net-map

Node number	Job role	Node number	Job role
**1**	Assistant head (Wellbeing and Safeguarding)	**11**	Learning and wellbeing department manager
**2**	Assistant head (PSE)	**12**	Learning support assistant (LSA)
**3**	Head of student support	**13**	Student support team (LSAs)
**4**	Head of PE	**14**	Canteen manager
**5**	PE teacher	**15**	Parent forum
**6**	Head of food technology	**16**	Anti-bullying team
**7**	Head of science	**17**	Wellbeing committee
**8**	Head of religious education	**18**	Transition key worker
**9**	Science teacher	**19**	Link governor
**10**	Parent/student support	**20**	Healthy Schools Coordinator
